# The association of calligraphy activities with peace of mind, stress self-management, and perceived health status in older adults

**DOI:** 10.3389/fpsyg.2024.1455720

**Published:** 2024-09-09

**Authors:** Jianing Wang, Kaizhi Tang

**Affiliations:** ^1^College of Art and Design, Hunan First Normal University, Changsha, China; ^2^Shanghai Academy of Fine Arts, Shanghai University, Shanghai, China

**Keywords:** calligraphy activities, peace of mind, stress self-management, perceived health status, older adults

## Abstract

**Introduction:**

Calligraphy, as a form of mindful practice, encourages focus, creativity, and relaxation, which collectively contribute to a more peaceful mental state. Through regular engagement in calligraphy, older adults can develop better coping mechanisms for stress, leading to more effective self-management of daily stressors. This enhanced ability to manage stress can reduce the overall burden on their mental and physical health, promoting a more positive outlook on life.

**Methods:**

This study employed convenience sampling and snowball sampling to select 246 older adults aged 60–70 from Changsha, China, in March 2024 as valid samples. AMOS v.23 was used to construct a structural equation model to validate the hypotheses.

**Results:**

The study found a significant positive correlation between calligraphy activities and peace of mind/stress self-management. There is also a significant positive correlation between peace of mind/stress self-management and perceived health status. Additionally, peace of mind and stress self-management act as mediators between calligraphy activities and perceived health status.

**Discussion:**

This indicates that calligraphy activities not only contribute to the psychological well-being of older adults but also indirectly enhance their positive perception of their own health by improving their mental state. Consequently, such activities can be an integral part of holistic health interventions aimed at enhancing the quality of life and overall health of older adults.

## 1 Introduction

According to data from the National Bureau of Statistics of China, as of the end of 2023, the population aged 60 and above reached 296.97 million, accounting for 21.1% of the total population. Among them, the population aged 65 and above was 216.76 million, comprising 15.4% of the total population (Xiao et al., 2024). Furthermore, projections indicate that by around 2035, older adults’ population aged 60 and above will exceed 400 million, making up over 30% of the total population, marking China’s entry into a severe aging period. Notably, nearly 75% of older adults population suffers from chronic diseases, with 40 million older adults disabled or semi-disabled, highlighting concerns about the health status of China’s elderly population (Hu and Glavin, 2023). Particularly following the COVID-19 pandemic, numerous studies have underscored the severe health anxiety experienced by older adults due to COVID-19 (Ioannidis et al., 2023). Those aged 60 and above are considered a high-risk group, with their physical and mental fragility requiring increased attention and care compared to other age groups (Su et al., 2023).

In terms of mental health, over 20% of older adults population suffers from various mental disorders such as depression (Papageorgiou et al., 2023), psychological distress, anxiety, and suicidal tendencies (Franca et al., 2024; Wuttke et al., 2023). Compared to younger individuals, older adults with these mental disorders often experience difficulty concentrating, dizziness or fainting during periods of anxiety, and symptoms like nausea or diarrhea, leading to unbearable distress (Carletti et al., 2023; Luo et al., 2024). Personally, the negative emotions triggered by health anxiety can result in decreased quality of life, reduced happiness, disability, and even more severe physical and mental harm (Pasha et al., 2023; Wardhani et al., 2024). Previous research has shown that with population aging, social pressures related to health are increasing, manifested in heightened global disease burdens, increased demand for medical services (Liu Q. et al., 2023), and rising mortality rates (Razimoghadam et al., 2023). Thus, there is an urgent need for effective interventions that can mitigate these mental health challenges and enhance the overall well-being of older adults.

However, despite numerous studies exploring mental health and coping strategies among older adults (Marziali et al., 2008; Webb and Chen, 2022), most research still predominantly focuses on pharmacological interventions (Pinquart and Duberstein, 2007; De Crescenzo et al., 2022), with relatively few studies examining non-pharmacological approaches (Chen et al., 2021). Artistic and creative activities, particularly calligraphy, as a traditional cultural practice, not only demonstrate unique advantages in maintaining mental health but also help older adults better manage daily stress by enhancing focus, emotional regulation, and self-expression. Research has shown that engaging in various creative and artistic activities can effectively improve the quality of life and overall happiness of older adults (Chiang et al., 2024; Lin et al., 2024). Among these activities, Chinese calligraphy stands out as a traditional cultural art form that integrates physical movement, meditation, and aesthetics (Shi, 2023). This unique combination is closely related to the mental health, stress management, and overall health status of older individuals. Practicing Chinese calligraphy involves a dynamic interplay of thoughts, body, and characters, requiring intense concentration, control, and coordinated movements (Yue et al., 2023). It stimulates sensory experiences and emotional expression, thereby aiding in stress relief, enhancing focus, and improving emotional regulation (Kao et al., 2021). For example, research has found that calligraphy can successfully enhance spatial abilities, visual attention, and image memory in patients with Alzheimer’s disease (Yan et al., 2022). Additionally, the aesthetic qualities of calligraphy can evoke feelings of pleasure and accomplishment, boosting confidence and life satisfaction among elderly practitioners (Yang et al., 2010). Therefore, in-depth research into the impact of calligraphy activities on the physical and mental health of older adults holds significant theoretical and practical importance.

Despite the potential benefits of calligraphy on the physical and mental health of older adults, existing literature primarily focuses on its aesthetic and creative aspects, with relatively few studies examining its therapeutic effects on psychological well-being, stress management, and overall health status (Han et al., 2024; Li et al., 2022; Ye et al., 2021). While some research has explored the psychological mechanisms of calligraphy practice, such as enhancing attention and emotional expression (Kao et al., 2021; Hsiao et al., 2023), there is a lack of systematic studies that demonstrate how these mechanisms translate into tangible health benefits for older adults. Additionally, current studies tend to emphasize pharmacological interventions, leaving a gap in understanding the role of alternative activities like calligraphy in promoting healthy aging (Christopher et al., 2023; Liu Y. et al., 2023; Okuyan et al., 2021). Although the value of non-pharmacological interventions in improving the health of older adults is increasingly recognized, most related research remains qualitative, lacking the quantitative evidence needed to support the broader application of calligraphy (Chang et al., 2024; Tripathi et al., 2022). Therefore, this study aims to fill this research gap by systematically investigating the impact of calligraphy activities on the psychological well-being, stress management, and perceived health status of older adults through quantitative methods.

The primary aim of this study is to explore the potential benefits of calligraphy activities for older adults, focusing on their impact on peace of mind, stress self-management, and perceived health status. Using quantitative research methods, this study analyzes how participation in calligraphy can enhance the psychological well-being of elderly individuals, improve their stress management capabilities, and influence their overall health perceptions. By addressing this research gap, the study aims to provide empirical support for incorporating calligraphy into health promotion strategies for older adults and to identify the specific mechanisms through which calligraphy exerts its beneficial effects. The innovation of this study lies in its systematic investigation of the relationships between calligraphy activities and the psychological health, stress management, and perceived health status of older adults. Unlike existing literature, which typically focuses on the aesthetic and creative aspects of calligraphy, this research emphasizes its practical health benefits. By employing quantitative methods to evaluate the effects of enhance our understanding of the role traditional calligraphy plays in promoting positive aging. The findings will provide valuable insights for policymakers and practitioners, encouraging the support of traditional cultural activities like calligraphy among older adults, thereby effectively enhancing their mental health, reducing levels of anxiety and depression, and ultimately improving their overall quality of life.

The paper is organized into the following sections: Section 2 outlines the hypotheses and conceptual models. Section 3 details the methods used for data collection and analysis. Section 4 presents the results of the data analysis and tests the proposed hypotheses. Section 5 provides a discussion, including theoretical contributions, practical implications, and limitations, along with suggestions for future research. Finally, Section 6 offers the conclusion.

## 2 Literature review and hypothesis development

### 2.1 Leisure theory

Leisure theory explores the significance and impact of leisure activities in human life (Rojek, 1997; Rojek, 2001; Isoahola et al., 1994). Introduced the concept of ‘serious leisure,’ emphasizing that some leisure activities require long-term commitment and professional skills (Stebbins, 2005; Stebbins, 2008). Participants in such activities gain a sense of achievement and self-fulfillment, which benefits personal development. Iso-Ahola and Mobily (1980) discussed leisure from a motivational perspective, suggesting that people engage in leisure activities to escape daily stress and to seek psychological pleasure and self-realization. These activities meet intrinsic needs, thereby enhancing subjective well-being. Overall, leisure theory focuses on meaningful and enjoyable activities pursued in free time, and how these activities influence mental and physical health and quality of life (Burnett-Wolle and Godbey, 2007). Leisure activities provide individuals with a space for autonomous, intrinsically motivated, and meaningful self-expression, fulfilling the need for self-actualization (Young Tae, 2017).

Calligraphy is an art form involving the elegant and aesthetic writing of characters using brushes, ink, paper, or other tools. It is not only a writing technique but also a means of expressing culture, emotions, and spirit (Woo, 2020). Calligraphy has a long history in many cultures, especially in East Asian countries like China, Japan, and Korea (Shin, 2017). From the social dimension of leisure theory, calligraphy offers a platform for social interaction and community building (Aung et al., 2022). Participating in calligraphy classes or workshops allows older adults to establish new social connections and strengthen existing ones (Rhee, 2019). Additionally, calligraphy can bring together individuals with a shared interest in the art form, fostering a sense of community and collective identity (Wu et al., 2024). Through calligraphy activities, older adults can enjoy personal achievements and receive support and encouragement from social interactions.

From the perspective of serious leisure in leisure theory, the practice of Calligraphy involves strict standards and requirements, such as copying classics and mastering techniques. Through long-term practice and dedication, older adults can gain a sense of achievement and self-fulfillment. Calligraphy provides a unique opportunity to slow down and engage in mindfulness practice, as the focus and calmness required in calligraphy practice can help reduce anxiety and stress. By applying Rojek’s leisure theory to the study of calligraphy activities, we can gain a deeper understanding of how this traditional art form promotes peace of mind, stress self-management, and perceived health status among older adults.

### 2.2 Hypothesis development

#### 2.2.1 Calligraphy activities, peace of mind, and stress self-management

Calligraphy, with its unique blend of artistic expression and meditative qualities, has been shown to significantly contribute to achieving a state of peace of mind (Choi, 2019). The intense focus required during calligraphy practice, where practitioners concentrate on each stroke and detail, mirrors the mindfulness seen in meditation practices, leading to reduced anxiety and a greater sense of tranquility (Zhang, 2023; Wu et al., 2024). These effects are not limited to East Asian contexts; research in other cultural settings has also suggested that engaging in similar mindful artistic activities can lead to reduced stress and enhanced emotional regulation (Unluer and Ozcan, 2010).

A peaceful state of mind is crucial in managing stress, as it allows individuals to approach life’s stressors with calmness, thereby adopting more positive coping strategies and enhancing their stress self-management abilities (Chen et al., 2012). The meditative process of calligraphy not only promotes peace of mind but also indirectly improves individuals’ abilities to manage stress. This is consistent with research on mindfulness practices, which have been shown to increase self-regulation and stress management by fostering focused attention, emotional regulation, and goal pursuit (Kao et al., 2021).

Additionally, a peaceful psychological state enhances the mobilization of internal resources to effectively respond to external pressures (Mao, 2022). When individuals are calm, they are more likely to adopt effective coping strategies, reducing negative reactions caused by stress (Usman et al., 2020). This relationship between peace of mind and stress management has been documented across different cultures, suggesting that the benefits of calligraphy and similar practices are not limited to East Asian traditions.

Therefore, this study proposes the following hypotheses:

H1: *Calligraphy activities have a positive and significant association with peace of mind.*

H2: *Calligraphy activities have a positive and significant association with stress self-management.*

H3: *Peace of mind has a positive and significant association with stress self-management.*

#### 2.2.2 Peace of mind, and stress self-management and perceived health status

Existing research indicates that psychological well-being, characterized by emotional stability (Yao, 2020), low levels of negative emotions, and high life satisfaction, is often associated with higher levels of perceived physical health (Lee et al., 2022; Zaninotto and Steptoe, 2023). When individuals are in a stable and peaceful psychological state, they tend to have a more positive evaluation and experience of their health status. Peace of mind refers to a state of calmness and psychological stability that can reduce anxiety and tension, leading to greater psychological comfort (Sun et al., 2024). When people are in a state of peace of mind, they are likely to view their health more positively, experiencing a sense of harmony and balance between mind and body (Xie, 2023).

Stress self-management involves the ability of individuals to effectively use various strategies to regulate and control their stress levels when facing life’s pressures (Zhang et al., 2024). Effective stress management can reduce the negative psychological and physiological impacts, thereby enhancing individuals’ positive perceptions of their health (Hong and Cho, 2021). When individuals effectively manage and cope with stress, they are more likely to experience physical well-being and mental happiness (Shepardson et al., 2017). Effective stress management enhances one’s ability to face challenges and promotes the proper functioning of physiological systems. Research shows that individuals with strong stress self-management skills tend to have a more positive perception of their health status (Xie et al., 2020).

Therefore, this study proposes the following hypotheses:

H4: *Peace of mind has a positive and significant association with perceived health status.*

H5: *Stress self-management has a positive and significant association with perceived health status.*

#### 2.2.3 Mediation effects

Engaging in calligraphy allows older adults to enter a meditative and relaxed state, focusing on the writing process, which alleviates anxiety and stress, thereby enhancing their sense of peace of mind (Liang et al., 2020). As hypothesized, peace of mind significantly enhances an individual’s perceived health status, a relationship that is supported by literature on mindfulness and meditative practices across various cultural contexts. This suggests that the positive impact of calligraphy on perceived health status is mediated by the state of peace of mind.

Moreover, calligraphy requires a high level of attention and control, which not only helps divert attention from stress but also cultivates stress management strategies and abilities among older adults (Zhang et al., 2024). The stress management skills developed through calligraphy are consistent with findings from other mindfulness-based interventions that show similar health benefits across different populations (Guo et al., 2019). Thus, stress self-management serves as a mediating variable, enabling the positive impact of calligraphy activities on perceived health status to manifest.

Drawing from literature on meditation, mindfulness practices, and stress management techniques, it is reasonable to propose that peace of mind and stress self-management are mechanisms through which participation in calligraphy activities influences older adults’ perceived health status (Al-Dwaikat et al., 2021; Xie, 2023). By fostering peace of mind and enhancing stress coping abilities, calligraphy activities may contribute to more positive health perceptions, encompassing physical, psychological, and emotional dimensions (Sun et al., 2024). This hypothesis integrates insights from research on mindfulness, meditation, and stress management, highlighting the potential of calligraphy as a holistic approach to enhancing overall health and well-being in older adults.

Thus, this study proposes the following hypothesis:

H6: *Peace of mind and stress self-management mediate the relationship between calligraphy activities and perceived health status.*

**Figure 1:**
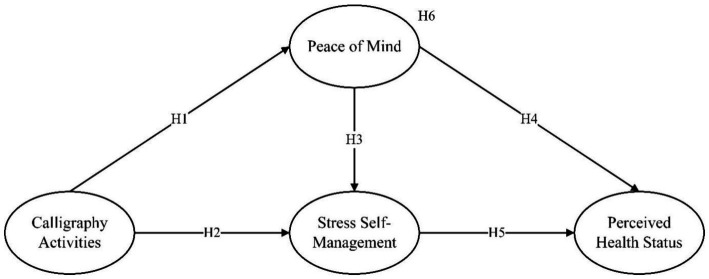
Hypothesis model.

All hypotheses are summarized in Figure 1.

## 3 Methodology

### 3.1 Participants and procedures

The subjects of this study are older adults aged 60–70 years because individuals in this age group are typically already retired and face changes in health, social relationships, and lifestyle. Researchers conducted a paper-based questionnaire survey among older adults aged 60–70 in Changsha, China, in March 2024. The survey employed convenience sampling and snowball sampling methods, which, while efficient for reaching the target population, may introduce certain biases and limit the generalizability of the results. However, these methods were chosen because they allow for efficient data collection within a specific community context, particularly when access to the target population is limited. Convenience sampling enabled quick access to a relatively large sample, while snowball sampling helped engage participants who might otherwise be difficult to reach, thus increasing the overall participation rate. Despite the potential for over-representation of more accessible individuals and the homogeneity that may result from relying on social networks, these methods were effective in capturing a representative snapshot of the older adult population in the selected communities.

The researchers contacted the residents’ committees of 12 communities in Changsha to help distribute the questionnaires within the communities. All participants were informed of the survey’s purpose and participated voluntarily before filling out the questionnaire. A total of 300 questionnaires were distributed, and 286 were returned. After excluding invalid questionnaires, 246 valid questionnaires were obtained, with an effective response rate of 82%.

Table 1 presents the demographic characteristics of the 246 older adults aged 60–70 who participated in this survey. Among the respondents: (1) 61.4% of participants were female; (2) most participants were aged 63–65 (accounting for 33.3%); (3) the vast majority (83.3%) of participants were not living alone; (4) over 70% of participants had an educational background of high school/vocational college/bachelor’s degree; (5) nearly half (42.7%) of participants had a monthly pension income between 3,001 and 6,000 CNY.

**Table 1:** Demographic characteristics (*n* = 246).

Profiles		*n* (%)
Gender	Male	95 (38.6)
	Female	151 (61.4)
Age	60–62	61 (24.8)
	63–65	82 (33.3)
	66–68	49 (19.9)
	69–70	54 (22.0)
Living alone status	Yes	41 (16.7)
	No	206 (83.3)
Education level	Junior high school and below	50 (20.3)
	High school/vocational college	87 (35.4)
	Bachelor’s degree	98 (39.8)
	Master’s degree and above	11 (4.5)
Monthly pension income	3,000 CNY and below	49 (19.9)
	3,001–6,000 CNY	105 (42.7)
	6,001–10,000 CNY	62 (25.2)
	Above 10,000 CNY	30 (12.2)

### 3.2 Instruments

The questionnaire comprised five sections. The first section requested respondents to provide their demographic information, including gender, age, living alone status, education level, and monthly pension. The second section utilized three items to collect data on respondents’ participation in calligraphy activities over the past week. The items included “How frequently did you practice writing last week?,” “How frequently did you learn new calligraphy techniques last week?,” and “How frequently did you attend calligraphy workshops or courses last week?.” The third section employed five items from the scale revised by Lee et al. (2013) to gather data on respondents’ peace of mind. Sample items included “I feel content and comfortable with myself in daily life.” The fourth section used six items from the scale by Pinar et al. (2009) to collect data on respondents’ stress self-management. Sample items included asking respondents about the frequency of actions such as “Take time for relaxation.” The fifth section utilized three items from the scale developed by Fernández-Jiménez et al. (2024) to compile data on respondents’ perceived health status. Sample items included “How would you rate your overall current state of health?”

To ensure the accuracy and cultural relevance of the questionnaire, the original scales, which were in English, underwent a professional translation process into Chinese. The translation followed a rigorous back-translation procedure to maintain conceptual equivalence across languages. First, two bilingual experts independently translated the scales from English to Chinese. These translations were then reviewed and synthesized into a single version by a third expert. Subsequently, a different pair of bilingual experts, who were blind to the original English versions, translated the Chinese version back into English. This back-translated version was compared with the original English text to identify any discrepancies. Any differences were discussed and resolved through consultation with both the translators and the research team to ensure that the final Chinese version accurately reflected the intent and meaning of the original scales while being culturally appropriate for the respondents. All four scales were measured using a Likert five-point scale, with response options ranging from 1 (Strongly Disagree, Never, Very Poor) to 5 (Strongly Agree, Always, Very Good).

### 3.3 Data analysis

In this study, AMOS v.23 was used to construct a structural equation model (SEM) to explore the relationships between calligraphy activities, peace of mind, stress self-management, and perceived health status. AMOS was chosen due to its user-friendly interface and robust capabilities for handling complex models and mediating effects, making it well-suited for SEM analysis. Additionally, AMOS supports graphical modeling, which facilitates clearer visualization and interpretation of relationships between variables. The maximum likelihood (ML) method was used to estimate the model parameters, following a two-step approach to evaluate measurement and structural models. Specifically, the measurement model was tested first to assess the reliability and validity of the constructs, followed by the evaluation of the structural model to examine the hypothesized relationships. The model’s reliability, validity, fit indices, path coefficients, and mediating effects were assessed.

To mitigate common method variance (CMV) from self-reported behaviors, Harman’s single-factor test was employed initially, followed by a comparison between two models using the chi-square difference test, as recommended by Mossholder et al. (1998). This test helps identify whether a single factor is responsible for the majority of the variance, reducing concerns about CMV. Model one’s chi-square value was 1660.507 with 135 degrees of freedom and a *p*-value below 0.001, while model two’s chi-square value was 333.494 with 113 degrees of freedom and a *p*-value below 0.001. These findings confirm the models’ fit, indicating that CMV is not a concern in this study.

## 4 Results

The following sections present the results of the measurement and structural models, using SEM to analyze the relationships between observed and latent variables. SEM is a widely used method in social sciences for testing complex theoretical models, allowing for the examination of both direct and indirect effects. In this study, confirmatory factor analysis (CFA) was employed to assess the reliability and validity of the measurement model, while the structural model was tested using AMOS v.23. This software was selected for its ability to handle complex models and mediating effects, providing a comprehensive analysis of how calligraphy activities influence psychological outcomes and perceived health in older adults.

### 4.1 Measurement model

The reliability and validity of latent variables were assessed using CFA with AMOS v.23. All variables had Cronbach’s *α* values above 0.8 (see Table 2), indicating strong internal consistency, as recommended by Fornell and Larcker (1981). The average variance extracted (AVE) for each variable was over 0.5, and the composite reliability (CR) exceeded 0.8, confirming the model’s convergent validity. Principal component factor analysis showed factor loadings between 0.701 and 0.955 (refer to Table 2), supporting the measurement model’s construct validity. Discriminant validity was demonstrated by the square root of AVE being higher than the correlations between constructs (see Table 3).

**Table 2:** Reliability and validity.

Items	Factor loadings	Cronbach’s *α*	CR	AVE
***Calligraphy activities (CA)***		0.914	0.916	0.784
CA1	0.893			
CA2	0.919			
CA3	0.843			
***Peace of mind (POM)***		0.954	0.955	0.811
POM1	0.781			
POM2	0.904			
POM3	0.955			
POM4	0.911			
POM5	0.940			
***Stress self-management (SSM)***		0.897	0.899	0.597
SSM1	0.701			
SSM2	0.773			
SSM3	0.797			
SSM4	0.747			
SSM5	0.823			
SSM6	0.788			
***Perceived health status (PHS)***		0.934	0.937	0.832
PHS1	0.949			
PHS2	0.922			
PHS3	0.864			

**Table 3:** Pearson correlation.

Construct	CA	POM	SSM	PHS
CA	(0.885)			
POM	0.393**	(0.901)		
SSM	0.427**	0.594**	(0.773)	
PHS	0.415**	0.712**	0.664**	(0.912)

The square root of the AVE is in diagonals; off diagonals are a Person’s corrections of contracts. **p < 0.01.

### 4.2 Structural model

**Figure 2:**
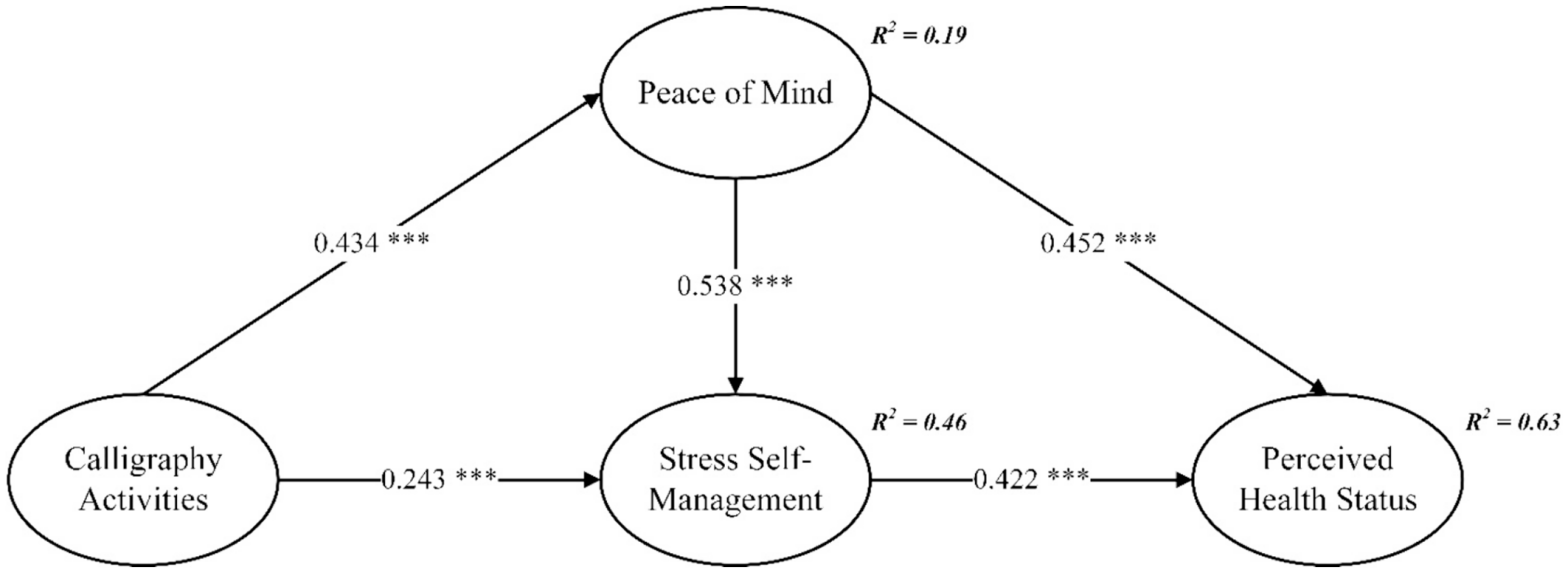
Structural path model. ***p < 0.001.

After thoroughly evaluating the measurement model’s reliability and validity, the study proceeded to analyze the structural model using AMOS v.23 to test the proposed hypotheses. AMOS was chosen due to its capabilities in SEM, particularly its robust handling of bootstrapping techniques for testing mediation effects. The model fit indices (χ^2^/df = 2.937, NFI = 0.917, IFI = 0.944, TLI = 0.922, CFI = 0.943) indicated a strong fit between the model and the empirical data. Pearson correlation results in Table 3 confirmed the interrelationships among variables. Figure 2 visually presents the standardized coefficients within the structural equation model.

As shown in Figure 2, calligraphy activities were positively correlated with peace of mind (*β* = 0.434, *p* < 0.001) and stress self-management (*β* = 0.243, *p* < 0.001), supporting hypotheses H1 and H2. Additionally, peace of mind positively correlated with stress self-management (*β* = 0.538, *p* < 0.001), supporting H3. Peace of mind also positively correlated with perceived health status (*β* = 0.452, *p* < 0.001), confirming H4. Finally, stress self-management was positively correlated with perceived health status (*β* = 0.422, *p* < 0.001), supporting H5.

### 4.3 Mediation effect analysis

Based on the results of the chain mediation effect analysis, this study examined three mediation paths involving the mediating variables. Using the Bootstrap sampling test method in the PROCESS plugin, with 5,000 samples, the results showed that the 95% confidence intervals of all paths did not include the number 0, indicating that all mediation effect paths were significant (see Table 4).

**Table 4:** Standardized indirect effect.

	Point estimate	Product of coefficients		Bootstrapping		
				Bias-corrected 95% CI		Two-tailed significance
		SE	*Z*	Lower	Upper	
CA → POM → PHS	0.134	0.037	3.590	0.118	0.262	*p* < 0.000
CA → SSM → PHS	0.057	0.025	2.294	0.035	0.132	*p* < 0.000
CA → POM → SSM → PHS	0.050	0.017	2.838	0.039	0.106	*p* < 0.000

Specifically, in the path “CA → POM → PHS,” the estimated effect value was 0.134, with a standard error of 0.037 and a confidence interval of [0.118, 0.262]. The significance test result was *Z* = 3.590, *p* < 0.001. This suggests that peace of mind significantly mediates the relationship between calligraphy activities and perceived health status. Next, for the path “CA → SSM → PHS,” the estimated effect value was 0.057, with a standard error of 0.025 and a confidence interval of [0.035, 0.132]. The significance test result was *Z* = 2.294, *p* = 0.022. This indicates that stress self-management also plays a significant mediating role between calligraphy activities and perceived health status.

In the chain mediation path “CA → POM → SSM → PHS,” the estimated effect value was 0.050, with a standard error of 0.017 and a confidence interval of [0.039, 0.106]. The significance test result was *Z* = 2.838, *p* = 0.005. These results demonstrate that the combination of peace of mind and stress self-management significantly mediates the relationship between calligraphy activities and perceived health status.

In conclusion, these findings support the hypothesized mediation effect paths in this study, indicating that calligraphy activities indirectly influence perceived health status through peace of mind and stress self-management, either individually or in combination. This supports Hypothesis 6. These results further clarify the complex relationships among the variables and contribute to a more comprehensive understanding of the phenomena explored in this study.

## 5 Discussion

### 5.1 Theoretical contributions

First, this study expands the application of leisure theory, particularly as articulated by Rojek (2001), by demonstrating how calligraphy, as a constructive leisure activity, positively impacts mental health and health perceptions. This research supports the notion that leisure activities contribute to personal development, cultural enrichment, and social integration, showing that calligraphy can foster personal growth, cultural connection, psychological well-being, and perceived health status in older adults.

Second, this study enriches the existing literature on the psychological benefits of traditional art forms (Martin-Wylie et al., 2024). While previous research often focused on the aesthetic and creative value of calligraphy, this study emphasizes its therapeutic potential. By systematically examining the mediating roles of inner peace and stress self-management, this research provides a detailed understanding of how calligraphy promotes psychological calmness and effective stress management, thereby enhancing overall health perceptions.

Third, this study integrates insights from peace of mind and stress management theories to offer a comprehensive framework for understanding how calligraphy activities influence health outcomes (Liang et al., 2020). By demonstrating that inner peace and stress self-management mediate the relationship between calligraphy practice and perceived health status, this research highlights the importance of these psychological factors in enhancing health perceptions.

However, implementing calligraphy programs as a therapeutic intervention may face practical challenges. Resource availability, such as access to instructors and materials, may limit the widespread adoption of such programs. Additionally, participant engagement, especially among those unfamiliar with or uninterested in calligraphy, may present a barrier to its success. Addressing these challenges will be crucial for the practical application of calligraphy as a non-pharmacological intervention.

Finally, this study contributes to the growing body of research on non-pharmacological interventions for stress and health management among older adults (Chang et al., 2024; Tripathi et al., 2022). It provides empirical evidence supporting the inclusion of calligraphy in health promotion strategies, suggesting that engaging in calligraphy can effectively achieve mental relaxation and stress reduction. These findings can guide policymakers and practitioners in incorporating calligraphy and similar leisure activities into health promotion and preventive care strategies, thereby supporting the overall health of the aging population.

### 5.2 Practical implications

Considering the direct and positive impact of calligraphy activities on improving older adults’ peace of mind and stress self-management abilities, as well as their indirect effect on enhancing perceived health status, the following recommendations are proposed:

First, national sports and cultural departments should collaborate to integrate calligraphy into existing health promotion strategies for older adults. For example, calligraphy activities could be incorporated into community wellness programs, alongside physical exercise or mental health initiatives. Organizing regular calligraphy training classes and competitions can stimulate interest, while also serving as a platform for social interaction. The government should support these efforts by providing resources and venues for communities and nursing homes. Colleges and universities could also contribute by deploying professional instructors to lead community-based calligraphy workshops. Additionally, integrating psychological health assessments as part of the calligraphy activities will ensure their effectiveness is monitored and improved over time. This approach will help promote calligraphy nationwide, supporting the physical and mental health of older adults in a structured and sustainable manner.

Second, digital platforms and online communities offer innovative avenues to expand the reach of calligraphy programs. Given the growing use of technology among older adults, virtual calligraphy classes, video tutorials, and online competitions could provide wider access to these activities, especially for those with mobility limitations or living in remote areas. Online forums and social media platforms could also facilitate interaction and experience-sharing among participants, further enhancing their engagement. Collaborating with digital platforms could help make calligraphy a more accessible and scalable intervention. Community centers, in parallel, can continue organizing in-person sessions with professional instructors, but by supplementing these activities with online resources, the scope and impact of calligraphy interventions can be broadened.

Third, at the family level, members should actively encourage older adults to participate in calligraphy, providing necessary time, space, and emotional support. Families could even participate in calligraphy activities together, strengthening intergenerational bonds. Encouraging older adults to use calligraphy as a way to manage stress and emotions at home can significantly enhance its therapeutic benefits. Furthermore, involving family members in the process can create a more supportive environment, which may increase older adults’ sustained engagement in the activity.

Fourth, at the personal level, older adults should actively engage in calligraphy activities, making it a part of their daily life to enhance their psychological health and stress management abilities through practice. They should continuously learn and improve their calligraphy skills, participate in more calligraphy-related activities and competitions, and maintain their interest and enthusiasm for calligraphy. During practice, older adults should pay attention to their own psychological and health conditions, providing timely feedback to family, friends, and doctors, and making adjustments if necessary to ensure the effectiveness of the activities. Through active personal participation, older adults can better enjoy the psychological and physical benefits brought by calligraphy, improving their quality of life.

By combining in-person activities with digital platforms, fostering community and family support, and encouraging personal commitment, the potential of calligraphy as a non-pharmacological intervention for stress and health management can be maximized, making it an accessible and effective tool for promoting the well-being of older adults.

### 5.3 Limitations

Firstly, this study is a cross-sectional study that investigates the behaviors and psychological activities of older adults through self-reported questionnaires. It did not employ experimental intervention methods. A key limitation of self-reported data is the potential for response bias, as participants may overestimate or underestimate their behaviors or feelings, leading to inaccuracies in the data. The limitation of cross-sectional studies is that they cannot establish causality, only reflecting correlations between variables. Therefore, future research is recommended to adopt longitudinal study designs or experimental interventions to more accurately understand the causal effects of calligraphy activities on the psychological health and stress management of older adults through long-term follow-up studies or intervention experiments. To address the potential bias in self-reported data, future studies could incorporate objective measures, such as physiological or behavioral assessments, to validate self-reported outcomes.

Secondly, this study utilized convenience sampling and snowball sampling methods, which may introduce sampling bias. The use of convenience sampling, while practical for reaching participants efficiently, may limit the representativeness of the sample, as those who were more readily available or within certain social networks were more likely to be included. Snowball sampling further reinforces this potential bias by relying on participant referrals. It is important to acknowledge this limitation, as it may affect the generalizability of the findings. Future research should aim to employ more randomized or stratified sampling methods to ensure a more representative sample of older adults and reduce the risk of bias.

Thirdly, this study focused on the age group of 60–70 years old, considering that older adults in this age range generally have good literacy skills, making it easier to distribute questionnaires. Therefore, it did not consider older adults beyond this age range. This choice may limit the generalizability of the findings to older age groups. Future research should expand the age range to include those aged 70 and above, and even those over 80, to more comprehensively understand the impact of calligraphy activities on different age groups, especially those who may have literacy difficulties. Additionally, future studies should consider cultural and socioeconomic differences that may influence older adults’ engagement with calligraphy, to broaden the applicability of the findings across diverse populations.

## 6 Conclusion

In accordance with the research objectives, this study has highlighted the significant potential of calligraphy as a beneficial activity for older adults, particularly in enhancing peace of mind, stress self-management, and overall perceived health. By systematically examining the impact of calligraphy through quantitative research methods, this study not only fills a critical research gap but also contributes to the existing literature on non-pharmacological interventions for mental health in older adults. The findings suggest that calligraphy can play a pivotal role in fostering peace of mind and effective stress management among older adults. The practice of calligraphy involves focused attention, controlled movements, and emotional expression, all of which contribute to its therapeutic effects.

Moreover, the study underscores the importance of understanding the mechanisms through which calligraphy exerts its benefits, such as promoting mindfulness and enhancing self-efficacy in managing stress. This research contributes to the broader body of knowledge by demonstrating how traditional cultural activities, like calligraphy, can be incorporated into health promotion strategies, thereby enriching the theoretical understanding of leisure and its psychological benefits for older adults.

In conclusion, integrating calligraphy into health promotion programs offers a promising, non-pharmacological approach to support the holistic health of the aging population. The empirical insights gained from this research can inform policymakers and practitioners, encouraging the adoption of traditional cultural activities to improve the quality of life and well-being of older adults. Future research should explore the applicability of these findings across different cultural contexts to better understand the global therapeutic potential of calligraphy for older adults. By leveraging the therapeutic potential of calligraphy, we can contribute to the development of more comprehensive and effective health interventions that address the unique needs of older adults.

## Data Availability

The raw data supporting the conclusions of this article will be made available by the authors, without undue reservation.
